# Sonification of Genomic Data to Represent Genetic Load in Zoo Populations

**DOI:** 10.1002/zoo.21859

**Published:** 2024-09-04

**Authors:** Edward J. Martin, Samuel A. Speak, Lara Urban, Hernán E. Morales, Cock van Oosterhout

**Affiliations:** ^1^ Institute of Ecology and Evolution, School of Biological Sciences University of Edinburgh Edinburgh UK; ^2^ School of Environmental Sciences University of East Anglia, Norwich Research Park Norwich UK; ^3^ Natural History Museum London UK; ^4^ North of England Zoological Society Chester Zoo Chester UK; ^5^ Helmholtz AI Helmholtz Zentrum Muenchen Neuherberg Germany; ^6^ Helmholtz Pioneer Campus Helmholtz Zentrum Muenchen Munich Germany; ^7^ School of Life Sciences Technical University of Munich Freising Germany; ^8^ Center for Evolutionary Hologenomics, Globe Institute, Faculty of Health and Medical Sciences University of Copenhagen Copenhagen Denmark

**Keywords:** endangered species, gamification, genomics, pink pigeon, public engagement

## Abstract

Maintaining a diverse gene pool is important in the captive management of zoo populations, especially in endangered species such as the pink pigeon (*Nesoenas mayeri*). However, due to the limited number of breeding individuals and relaxed natural selection, the loss of variation and accumulation of harmful variants is inevitable. Inbreeding results in a loss of fitness (i.e., inbreeding depression), principally because related parents are more likely to transmit a copy of the same recessive deleterious genetic variant to their offspring. Genomics‐informed captive breeding can manage harmful variants by artificial selection, reducing the genetic load by avoiding the inheritance of two copies of the same harmful variant. To explain this concept in an interactive way to zoo visitors, we developed a sonification game to represent the fitness impacts of harmful variants by detuning notes in a familiar musical melody (i.e., Beethoven's *Für Elise*). Conceptually, zoo visitors play a game aiming to create the most optimal pink pigeon offspring in terms of inbreeding depression. They select virtual crosses between pink pigeon individuals and listen for the detuning of the melody, which represents the realised load of the resultant offspring. Here we present the sonification algorithm and the results of an online survey to see whether participants could identify the most and least optimal offspring from three potential pink pigeon offspring. Of our 98 respondents, 85 (86.7%) correctly identified the least optimal offspring, 73 (74.5%) correctly identified the most optimal, and 62 (63.3%) identified both the most and least optimal offspring using only the sonification.

## Graphical abstract

We developed a sonification algorithm that uses sound to communicate to zoo visitors the level of inbreeding of potential pink pigeon offspring. Listeners to the output of our algorithm were successful in completing a task identifying ‘good’ and ‘bad’ offspring from sound outputs alone.

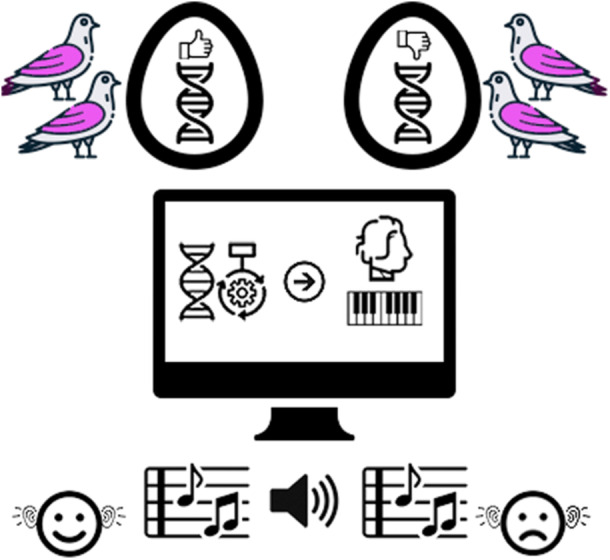

## 1 Introduction

Effective public engagement with research requires innovative approaches to communicate the often complex concepts and vast amount of data generated in science today. In the United Kingdom, the public audience tends to be disengaged with respect to science in general (Middleton et al. 2023). This is true for genomics in particular, a jargon‐rich and abstract branch of science that directly impacts many lives: in the United Kingdom one in 17 will be affected by a rare disease, most probably with a strong genetic component (HM Government 2020). Commonly, graphics are used to illustrate or conceptualise patterns in big data, including genomic sequencing data. However, such figures are often created for complex analytic tasks that each resist simplification (Rittenbruch et al. 2022), and can be difficult to interpret for people who are not trained to read data presented in such a format. Alternatively, rather than using visualisation, the information content of big data can be captured using sounds. By mapping some feature of the data to a sound synthesis parameter, a parameter‐mapping sonification can facilitate understanding in the listener (Grond et al. 2011). Previous work has seen success in communicating genomics using sonification techniques, for analytical (Martin, Meagher, and Barker 2021), communication (Temple 2021), and public engagement purposes (Plaisier, Meagher, and Barker 2021).

The role of zoos as sites of wildlife conservation is the basis of contemporary justification for their continued existence (Carr and Cohen 2011). Zoos are keen to present conservation credentials in detail to their public, while at the same time ensuring entertainment to attract sufficient visitors to maintain economic viability. Sonification has shown potential in being used to create public engagement activities which can convey scientific detail, while also achieving audience experiences with ‘wow‐factor’ (Ballora 2014).

Genomics techniques are increasingly used for wildlife conservation and management (Hohenlohe, Funk, and Rajora 2021). However, evolutionary concepts continue to be poorly integrated into conservation policies and management practices (Cook and Sgrò 2018). The mathematical complexity of quantitative genetics and population genetics, as well as the rapid advances of DNA sequencing technologies and bioinformatics, form real barriers that hinder the integration of conservation science into applied conservation (van Oosterhout 2021). With improved understanding of the causes of inbreeding depression, practitioners will be better able to manage small, captive populations in zoos. Also, as zoos are often public bodies, communicating the impacts of genetic management of zoo populations to visitors and other stakeholders is important.

In this proof‐of‐concept study, we present a sonification algorithm to help listeners to interpret the information that is present in a large quantity of DNA data using sound. In particular, our approach aims to explain how harmful genetic variants (e.g., deleterious mutations) reduce the fitness of individuals with parents that are related. We presented our sonification approach to an audience, detuning the pitch of a well‐known melody to communicate how genomic data can be used in conservation management to reduce inbreeding depression.

The loss of fitness due to inbreeding depression is a function of both the level of inbreeding and the genetic load of the individuals in the population (Crow 1970). The *genetic load* constitutes the deleterious fitness effects of harmful genetic variants that individuals carry in their genome (Bertorelle et al. 2022). The genetic load can be divided into two parts emphasising the effect of the load, with the *realised load* describing that which reduces the fitness in the current generation (Bertorelle et al. 2022). The importance of minimising inbreeding and maintaining genetic diversity is well established in the conservation community (Hoban et al. 2013; DeWoody et al. 2021). Pedigree data is widely used to manage captive populations to reduce the loss of variation and avoid close inbreeding (i.e., consanguineous mating) (Ballou and Lacy 1995; López‐Cortegano, Moreno, and García‐Dorado 2021). It has now also become possible to quantify the genetic load of individuals using genomics data and bioinformatics techniques (Bertorelle et al. 2022). The mutation‐impact scores of genetic variants can be estimated, for example using Combined Annotation Dependent Depletion scores (CADD scores) (see below). The quantification of the genetic load allows the impact of harmful mutations to be compared between all individuals with sequenced genomes. These advances considerably expand the possibilities for the management of captive‐bred populations, potentially reducing the severity of inbreeding depression (van Oosterhout 2020). Besides minimising the relatedness of individuals using pedigree‐calculated inbreeding coefficients, we can now establish whether individuals share harmful variants at the same genetic loci. By avoiding crossings between individuals with such a shared genetic load, it has become possible to reduce the realised load of the offspring caused by homozygous harmful variants. Theoretically, this could reduce inbreeding depression and improve the fitness of the offspring relative to that of a randomly mating zoo population, and zoo populations that are managed using pedigree‐data only (Speak et al. 2024). The analysis of the genetic load is increasingly performed in studies that analyse the genomes of threatened species (reviewed in Bertorelle et al. 2022), and they can help guide conservation management and predict the long‐term extinction risk (van Oosterhout 2020).

In a recent pilot study, which we use as the source of the data for our method, Speak et al. (2024) analysed the genetic load of pink pigeons (*Nesoenas mayeri*). The species recovered from a severe population bottleneck, before which an ex situ population was established (Jackson et al. 2022). Captive‐bred individuals were released into the wild population in Mauritius to increase population size and genetic variation. This conservation rescue program was very successful and resulted in population recovery (Jackson et al. 2022). Consequently, the pink pigeon was twice downlisted on the International Union for the Conservation of Nature (IUCN) Red List. Nevertheless, concerns remain because of its high genetic load (Jackson et al. 2022).

Our sonification approach is designed to enable zoo visitors to identify optimal mate pairs in zoo populations that produce the fittest offspring. Our application is gamified in the sense that the zoo visitors (i.e., players) are challenged finding the optimal crosses based on the minimal detuning in Ludwig van Beethoven's composition *Für Elise*. Digital games are increasingly used in teaching the value of biodiversity, and they have the potential to make a positive contribution to conservation (Sandbrook, Adams, and Monteferri 2015). In our ‘game’, deleterious genetic variants present in the genomic sequence data of the offspring of two birds cause the detuning of the melody. The purpose of the game is to minimise the detuning in the music and select the optimal breeding pairs to generate a viable population.

## 2 Material and Methods

Our sonification application maps genomics data to sound to allow the information present in the DNA to be interpreted through listening. The aspect of the genomic data that we are sonifying is the *reaslised* load, the aspect of the genetic load that reduces fitness in the current generation. Briefly, the potential fitness impacts of deleterious variants are first estimated, and these effects are then separated into two components: the masked load and the realised load (Bertorelle et al. 2022). The masked load is present only at heterozygous loci with harmful variants that are (partially) recessive. This part of the load does not reduce the fitness of its carrier. In contrast, the realised load does reduce fitness, and it is present at homozygous loci with harmful variants. In addition, some heterozygous loci with partially dominant harmful variants also contribute to the realised load. Furthermore, hemizygous loci with harmful variants in the sex chromosomes also add to the realised load, but only in the heterogametic sex (i.e., males in mammals, and females in birds).

In small populations, the masked load becomes converted into a realised load due to inbreeding and drift (Dussex et al. 2023). Genomics‐informed captive breeding can help minimise the realised load by crossing parents that do not share harmful variants at the same loci. To accomplish this, Speak et al. (2024) calculated the expected realised load of potential offspring of all possible crossings between the six pink pigeons that had been genome sequenced. Virtual crosses were produced, and the multilocus genotypes of the offspring were established assuming Mendelian segregation. The predicted realised load of potential offspring is a proxy for the expected amount of inbreeding depression. This approach enabled Speak et al. (2024) to identify optimal mate pairs that are expected to produce offspring with the lowest realised load and the least amount of inbreeding depression.

The source data, and contextual information, for the method comes from the study by Speak et al. (2024). Speak et al. (2024) used CADD to assess the genetic load in six pink pigeons. CADD ranks the severity of mutations across the genome, and the CADD scores have been established for every possible nucleotide substitution in the genome of model species such as humans, mice, and chickens. Speak et al. (2024) calculated the CADD scores for 603 genetic variants at 4929 ultra‐conserved elements (UCEs) and their flanking regions in the six pink pigeons. The pink pigeon reference genome consists of 2133 scaffolds, and Speak et al. (2024) analysed the sequences of six birds, quantifying the genetic load in the 100 largest scaffolds (longest scaffold = 38,883,656 bp and shortest = 5370 bp; median size = 5,486,524 bp). Together, these 100 scaffolds represent 68.96% of the pink pigeon reference genome. There are 595 UCEs across these 100 scaffolds that possess 658 single nucleotide polymorphisms (SNPs). These SNPs have relatively high mutation‐impact scores, estimated by CADD scores. The realised load scores per scaffold, as measured by the summation of CADD scores within each scaffold, were produced using R (R Core Team 2021), tidyverse (Wickham et al. 2019), and the figures were produced using ggplot2 (Wickham 2016). For more detailed information about the approach including visual information about the DNA sequences, see Speak et al. (2024).

To examine the genetic compatibility of individuals, we calculated the CADD scores of the shared genetic load of deleterious variants between all different pairwise combinations of individuals (Speak et al. 2024). To illustrate the effect of extreme inbreeding, we also included ‘selfing crosses’ where an individual was able to reproduce offspring by self‐fertilisation or uniparental reproduction. Offspring of parents that share many deleterious variants at the same genetic loci are expected to inherit a high realised load of homozygous variants. Such offspring are likely to suffer from severe inbreeding depression.

To sonify the load, we have developed an auditory analogy relating the ‘wrongness’ of a familiar melody with the risk of deleterious mutations in the genome of a potential pink pigeon offspring. The realised load scores of an individual potential offsping, calculated by the summation of the CADD scores across each scaffold, detune a well‐known melody. The detuning of the pitch of a note represents the realised load score of an individual potential offspring at a corresponding locus, as calculated by the summation of CADD scores across a scaffold. This communicates the predicted relative fitness loss of the offspring. An alphabetical ordering of the scaffolds was used and is an artefact of the analysis. This ordering is consistent between different outputs and facilitates comparison, with size or scaffold location unused alternatives. To detune a familiar western classical melody, we used the musical instrument digital interface (MIDI) protocol as a method for manipulating sound. MIDI is a language based on western classical music tonality that allows the real‐time control of sound synthesis and remains an industry standard. To create the effect of detuning individual notes, we made use of the pitch‐bend functionality of MIDI. However, rather than varying the pitch of a note dynamically while it is played as in *glissando*, *portamanto*, or the use of pitch‐bend wheels, we used the functionality to give detuned notes that were off‐key but unvarying as the note sounded, closer to the idea of *blue notes* in jazz or folk music. MIDI represents pitch‐bend as a 14‐bit integer, allowing for 16,384 values with −8192 representing maximum downward bend, 8192 representing maximum upwards bend, and 0 representing no bend. We used the default maximum bend as ±2 semitones.

The realised load scores of our data range between 0 and 376. We made two transformations to these to give MIDI pitch‐bend values. First, we rounded them to the nearest factor of five to obtain integer values and to not detune the note in the case of low genetic loads. Second, we multiplied these values by −20 to obtain only negative values, representing downward bend or ‘detuning’ in our analogy, and to render the detuning more distinguishable. The range of values between 0 and −7520 resulted in an appropriate level of ‘detuning’. We then used Python version 3.7.4 (van Rossum and Drake 2009) and Jupyter Notebook version 6.0.1 (Kluyver 2016) for data analysis. We used the mido package (v1.2.6) to read and write MIDI data. We used the pandas package (v0.25.1) to read our csv‐formatted genomic data (sum of CADD scores of 100 largest scaffolds of 36 resultant crosses from six birds), and also to create a list of strings to manipulate the MIDI messages. We used pygame (v2.0.1) to play the output MIDI file in the Jupyter Notebook. We created wave file (.wav) examples of the sonifications using Ableton 10 Live Suite.

The sonification algorithm requires a single‐channel MIDI track as the basis for detuned sonification outputs. We used Musescore 3 (v3.6.2) to transcribe a version of the well‐known Ludwig van Beethoven composition *Für Elise* (see Supporting Information Materials). The version we created includes 104 notes, of which we used 100 notes to be detuned according to the realised load scores of the 100 largest pink pigeon genome scaffolds. We opted to allow the first four notes of the melody to have no detuning—this gives the listener a brief frame of reference to aid in the perception of detuning throughout the rest of the sonification.

Programmatically, the algorithm uses mido to read the MIDI files. It then parses them into a list of strings and uses string methods to edit the MIDI messages. A loop, indexed through the list, alters the data by adding a MIDI *pitchbend* message preceding each *note_on* message which indicates the onset of a note. This MIDI *pitchbend* parameter is set to the value of the *i*th scaffold realised load score of the parental selection. It is worth noting that *note_on* messages are also used to end notes by setting the velocity parameter to 0, so our algorithm checks for a nonzero velocity of the *note_on* message. The algorithm also ignores the first four note onset messages, leaving an initial run of four unchanged notes at the start of each output sonification. After the completion of this loop, the list of strings is parsed by means of another loop so mido can save it as a new MIDI file. The algorithm is available as a Jupyter notebook at https://github.com/sonifyed/pinkpigeons.

To test whether our sonification algorithm was successful in communicating genetic load, we created a short experiment via a JISC online survey (see Supporting Information Materials). Participants were given access to audio recordings of the sonification representations of three pink pigeon potential offspring: ‘Offspring A’ (https://soundcloud.com/sonifyed/pink-pigeons-offspring-a), ‘Offspring B’ (https://soundcloud.com/sonifyed/pink-pigeons-offspring-b), and ‘Offspring C’ (https://soundcloud.com/sonifyed/pink-pigeons-offspring-c). Participants were also given access to a version of *Für Elise* without any detuning for comparison (https://soundcloud.com/sonifyed/fur-elise).

After a short introduction, providing some context about the pink pigeon and some explanation of how to listen to the sonifications, participants were asked *By listening to the sound, can you determine which is the most optimal offspring with the least detuning of the melody, and which the least optimal offspring with the most detuning of the melody?* This gave us a ranking of the three offspring from most to least optimal for each participant. We also offered a free text box prompted by the question *Do you have any comments you wish to share?* A copy of the survey can be found in the supplementary materials. Our participants were principally recruited via mailing lists at the University of Edinburgh. The survey was subject to ethics approval from the University of Edinburgh's SBS Ethics Committee with reference dbarker‐0004.

## 3 Results

**Figure 1:**
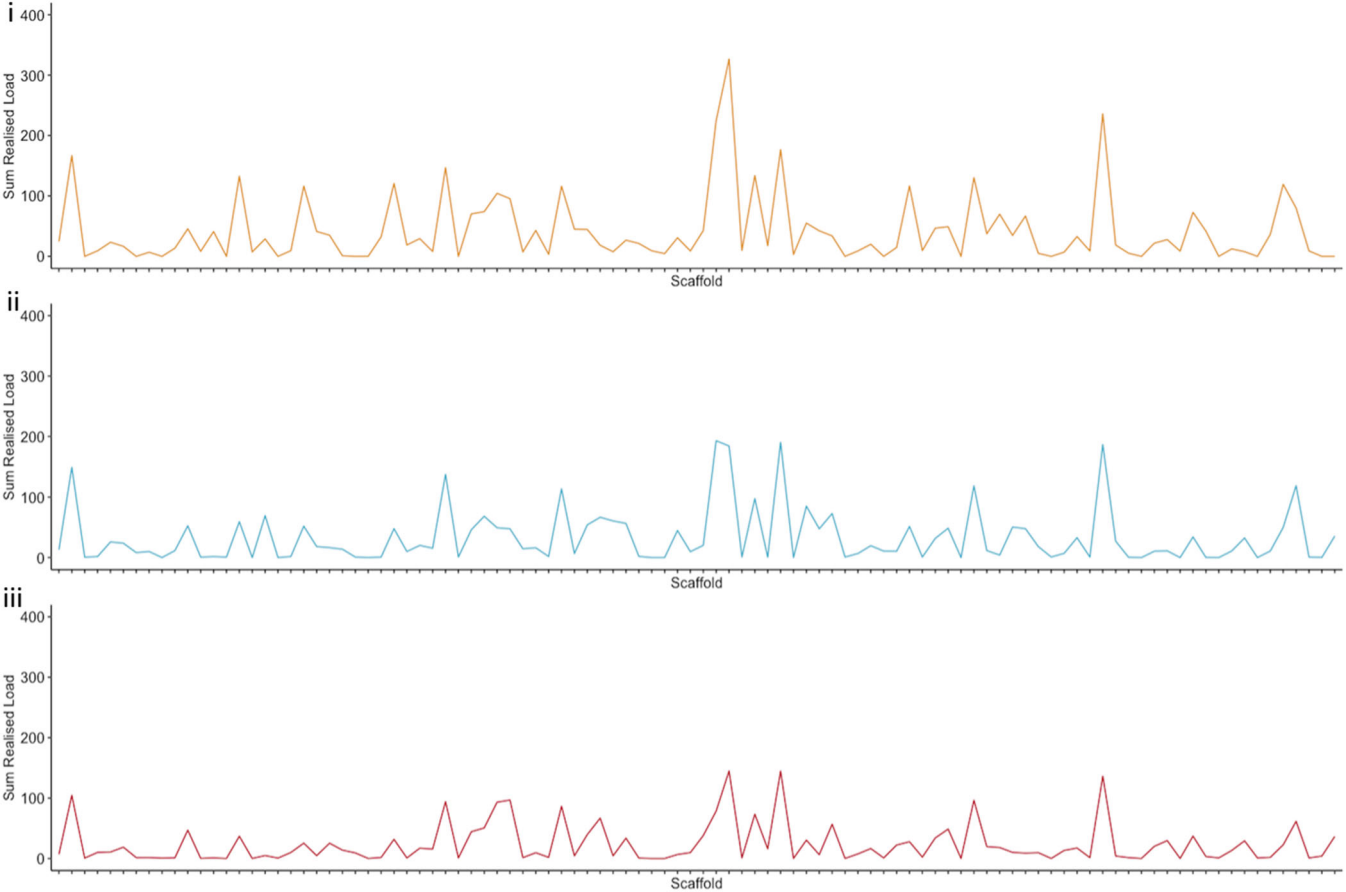
The realised load across the 100 largest scaffolds in offspring produced by crossing different combinations of pink pigeons. Panel i shows a selfing‐cross PP1 × PP1, resulting in a very high realised load (‘Offspring B’ with corresponding sonification: https://soundcloud.com/sonifyed/pink-pigeons-offspring-b). Panel ii is cross PP2 × PP3, which also produces offspring with a high realised load (‘Offspring A’ with corresponding sonification: https://soundcloud.com/sonifyed/pink-pigeons-offspring-a). Panel iii is cross PP2 × PP6, which results in offspring with a low realised load (‘Offspring C’ with corresponding sonification: https://soundcloud.com/sonifyed/pink-pigeons-offspring-c). The scaffolds were ordered alphabetically by their name, which does not correspond to size or location on the genome.

Figure 1 illustrates the realised load across the 100 largest scaffolds in offspring produced by crossing different combinations of pink pigeons. When an individual is crossed with itself, the realised load of the offspring is relatively elevated, as shown in the raised peaks in Figure 1, panel i. For example, the realised load of the offspring produced by the selfing cross PP1 × PP1 (i.e., ‘Offspring A’ https://soundcloud.com/sonifyed/pink-pigeons-offspring-a) is significantly higher compared to that of an outbred crosses PP2 × PP3 (i.e., ‘Offspring B’ https://soundcloud.com/sonifyed/pink-pigeons-offspring-b) (*T* test: *T* = 5.60, d.f. = 1225, *p* < 0.0001). Due to the small effective population size of the captive pink pigeon population, some individuals (e.g., PP2 and PP3) share a high genetic load. Crosses between such individuals produce offspring with a significantly higher realised load (Figure 1, panel i; https://soundcloud.com/sonifyed/pink-pigeons-offspring-b) than crosses such as PP2 × PP6 (i.e., ‘Offspring C’ https://soundcloud.com/sonifyed/pink-pigeons-offspring-c) whose parents share relatively few deleterious variants (Figure 1, panel iii) (*T* test: *T* = 2.68, d.f. = 1254, *p* < 0.0075). Offspring of the latter cross (PP2 × PP6) are expected to have a higher fitness, and this is reflected by the lower peaks in Figure 1, panel iii, and the relatively low level of detuning of the melody in the corresponding soundtrack.

In the sonification experiment, based on the answers of 98 respondents, the least optimal cross resulting in offspring with the highest realised load was correctly identified in 85 out of 98 cases (86.7%). The results are reported in Table 1. The most optimal cross was correctly identified in 73 out of 98 cases (74.5%). Both the most optimal and the least optimal were identified in 62 out of 98 responses (63.3%). These responses were significantly better than random, in a binomial test taking the null hypothesis of a random trial with π ≤ $\frac{1}{6},x=62,\;\mathrm{and}\;n=98;\;p<10^{-26}$. This provides evidence that our sonification method is successful in communicating the fitness of potential pink pigeon offspring when compared to random guessing.

The free text box prompted 18 responses (18.4% of participants). Six people stated that the task was difficult with two of them citing auditory impairments as a cause, five expressed that they found joy in the task, five stated an opinion that a pair were difficult to distinguish (four said A and C were very similar, and one said A and B), and two people made jokes pitying the inbreeding of B. Of the six people (33.3% of free text responses) that cited the difficulty of the task, five of them got the correct answers for both the most and least optimal. Out of this group, the only one to give incorrect answers had identified themselves as having auditory impairment. Of the five that stated that a pair were difficult to distinguish, four gave the correct answers for both most and least optimal. Notably no‐one said that B and C were difficult to distinguish, the pair with the largest difference.

## 4 Discussion

In this proof‐of‐concept study, we used sonification to introduce to zoo visitors how genomic data can be used to optimise the fitness of offspring by identifying parents that share little genetic load at the same loci. We used data from Speak et al. (2024), who quantified the fitness impacts of harmful variants (i.e., deleterious mutations) across the genome using CADD scores, studying the genome data of six pink pigeons. The study made virtual crosses between these individuals and calculated the realised load of the potential offspring, assuming Mendelian segregation of alleles (see Speak et al. 2024). The offspring of parents that share recessive deleterious variants at the same genetic loci can inherit two copies of the same variant, which increases their realised load and may result in inbreeding depression (Bertorelle et al. 2022).

Genomic data is likely to play an increasingly important role in maintaining the long‐term viability of zoo populations (Norman, Putnam, and Ivy 2019; van Oosterhout 2020). The efficacy of natural selection to purge the genetic load might be compromised in zoos due to a variety of factors. First, the small breeding population size reduces the effective population size, which increases genetic drift, thereby reducing the efficacy of natural selection (Dussex et al. 2023). Second, the relatively benign environmental conditions in zoos can interfere with hard selection by allowing individuals that might have otherwise died in the natural environment to survive and reproduce in captivity (Armbruster and Reed 2005). Third, in pedigreed zoo populations, conservation management aims to breed from individuals that are genetically underrepresented in the population to maximise founder representation and founder genome equivalents (Lacy 1989). Although this helps to conserve genetic diversity, it could also interfere with natural selection and the purging of the genetic load that may occur in nature. Genomic analysis can detect harmful variants and reduce their impact by avoiding crosses of individuals that share the same variants. The purpose of this study is to introduce this concept to laypersons, in particular, zoo visitors.

Our results show success compared to random guessing, and give evidence that the sonification method successfully communicates the genetic load of the offspring. Interestingly, respondents who gave feedback that the task was difficult proved to be more successful in completing the task than those who gave no feedback. Perhaps this reflects that the task feels difficult, even while people show quite a high success rate. This is an interesting future idea to explore, how a challenging public engagement task is perceived by its audience, and how it impacts the effect it has on them.

Explaining the value of conservation genomics to the public in an understandable and playful manner can make a meaningful contribution to conservation teaching. Future research directions could involve the development of an in situ installation at a zoo to investigate the impact of the idea in its intended context. The future implementation of our sonification algorithm in a zoo context as a public engagement game may have multiple impacts: raising awareness of genomics research and research findings, meeting the conservation learning mission of zoos, mobilising changes in behaviour, and also providing feedback to improve the public engagement research approach. Also, the sound design of the sonification could be developed to make a more emotionally compelling experience. As far as we are aware, the approach to sonification by detuning the notes in a familiar melody to represent ‘wrongness’ or ‘distance from an ideal outcome’ is a novel one, and many new applications of this idea in different fields can be imagined.

Charles Darwin stated in *On the Origin of Species* ‘pigeons have been watched, and tended with the utmost care, and loved by many people’ (Darwin 1859). We hope our sonification work highlights and supports the continuation of this tradition in the genomics era.

**Table 1:** Survey results and sum of CADD scores across 100 largest scaffolds. A total of 98 responses are listed here. The CADD scores listed are the sum of CADD scores for the largest 100 scaffolds for each potential offspring, and they provide an estimate of the realised load expected in the offspring.

	Offspring A	Offspring B	Offspring C
‘Parent’ Pigeons	PP2 and PP3	PP1 and PP1	PP2 and PP6
‘Which is the most optimal offspring?’ (*n* = 98)	25 (25.5%)	0	73 (74.5%)
‘Which is the least optimal offspring?’ (*n* = 98)	11 (11.2%)	85 (86.7%)	2 (2%)
Sum of CADD scores (1 d.p.)	3318.9	4239.2	2390.1

## Author Contributions

E.J.M., S.A.S., and C.v.O. conceived the study and drafted the MS. E.J.M. created the sonification algorithm, sound files, questionnaire, and analysed results of the questionnaire. S.A.S. produced the figure, and C.v.O. performed the statistical tests. All authors contributed intellectually to the study, and commented and edited the manuscript.

## Ethics Statement

This research was approved by the Ethics Committee at the School of Biological Sciences, University of Edinburgh (Reference number dbarker‐0004). All methods were carried out in accordance with relevant guidelines and regulations. Regarding the genomic data, publicly available DNA sequencing data were analysed, and no animals were used in this research. The data used is available at https://github.com/sonifyed/pinkpigeons.

## Conflicts of Interest

The authors declare no conflicts of interest.

## Data Availability

All supplementary materials including our algorithm, input midi file, example questionnaire, and the data used to create our sonifications, are made available as a Jupyter Notebook available at https://github.com/sonifyed/pinkpigeons.
